# Supplementary material for: Barriers and enablers of implementing bubble Continuous Positive Airway Pressure (CPAP): Perspectives of health professionals in Malawi
Source: PLoS One. 2020 Feb 13;15(2):e0228915. doi: 10.1371/journal.pone.0228915 (PMC7018070; doi:10.1371/journal.pone.0228915)
Supplement: S1 Appendix — (DOCX) [file pone.0228915.s001.docx]

**Supplementary Information**

**Appendix: Codebook**

| **Nodes** | **Sub-nodes** |
| --- | --- |
| Training | - Discussions of training they underwent, who trained them, length of training, what did they learn - Level of expertise and years of experience - Informal or formal training - Comments regarding satisfaction or dissatisfaction with the level of training received - Anything they wished they could have learned |
| Initiation | - Issues around initiation of bubble CPAP - Decision-making – who make decisions to start the neonate on bubble CPAP including doctors, registars, bubble CPAP nurses, household decision makers (mothers, fathers, mothers-in-law); who is in principle supposed to make the decision around initiating bubble CPAP and what happens in reality - Initiating bubble CPAP before or after consulting with mothers/guardians - Discussions around legibility to initiate bubble CPAP - Disagreements on initiation of bubble CPAP and how it is resolved |
| Monitoring | - Issues around monitoring the neonate once bubble CPAP has been initiated - Comments regarding roles and responsibilities - Ideals for monitoring versus actual monitoring - Role of mothers/guardians in monitoring and what they are doing - Day shift versus night/weekend shift - Staff shortages |
| Perceptions of mothers/  guardians | - Issues of acceptability - Myths around bubble CPAP - Perceptions of efficacy or ineffectiveness - Values associated with bubble CPAP (i.e. such as seriousness of the condition, baby has crossed the line of no return, meaning of putting someone on oxygen as end of life procedure) - Any mention of feelings they have around bubble CPAP such as fear, worry, happiness, etc - Mentions of parents resisting bubble CPAP and reasons why (i.e such as lack of information, myths, etc.) |
| Perceptions of health care professionals | - Issues of acceptability - Myths around bubble CPAP - Perceptions of efficacy or ineffectiveness - Values associated with bubble CPAP (i.e. such as seriousness of the condition, baby has crossed the line of no return, meaning of putting someone on oxygen as end of life procedure) - Any mention of feelings they have around bubble CPAP such as nervousness, fear, trepidation, joy, etc. |
| Infrastructure/ supplies | - Discussions of what is available, what is not, what items are used - Issues around assembly of machine and/or setting up - Issues around scarcity of resources and what happens, such as hiding the bubble CPAP machine - Issues around electricity, oxygen concentrators, availability of CPAP machines etc. - Discussions around maintenance and functionality, items breaking down - If there is an infrastructure or supplies gap, what is done instead? |
| Daily living activities of neonates | - Discussions around how to assist mothers/guardians in caring for the daily needs of their infant, such as feeding and changing diapers - Discussions of any difficulties mothers/guardians face while caring for their neonate that is on bubble CPAP |
| CROSS CUTTING THEMES | |
| Challenges | - Challenge or barrier to bubble CPAP |
| Support and enablers | - Anything that supports the use of bubble CPAP - Who supports, how to support, peer support (i.e. mothers supporting each other) |
| Stories of benefits | - Narratives shared by interviewees about benefits of bubble CPAP, success stories - Descriptions on how success with bubble CPAP is measured |
| Stories of complications and difficulties | - Narratives shared by interviewees about complications and difficulties faced while using bubble CPAP |
| Case examples | - Any key or evocative case examples |
| Other | - Anything else interesting of note but does not fit into the existing nodes |
